# Association Between Patient-Prescriber Racial and Ethnic Concordance and Postpartum Pain and Opioid Prescribing

**DOI:** 10.1089/heq.2021.0130

**Published:** 2022-03-07

**Authors:** Nevert Badreldin, Julia D. DiTosto, William A. Grobman, Lynn M. Yee

**Affiliations:** Division of Maternal-Fetal Medicine, Department of Obstetrics and Gynecology, Northwestern University School of Medicine, Chicago, Illinois, USA.

**Keywords:** postpartum pain, postpartum opioid, pain management, racial concordance, racial disparities

## Abstract

**Objective:**

To evaluate whether patient-prescriber racial and ethnic concordance is associated with postpartum opioid prescribing patterns and patient-reported pain scores.

**Methods:**

This is a retrospective cohort study of patients who delivered at a tertiary care center between December 1, 2015 and November 30, 2016. Self-identified non-Hispanic white (NHW), non-Hispanic black (NHB), Hispanic, or Asian patients were included. Patient-prescriber pairs were categorized as racially and ethnically concordant if they shared the same racial and ethnic identity; the prescriber was defined as the obstetrical provider who was responsible for the postpartum discharge of the patient. Multivariable regression models controlling for demographic and clinical confounders were used to assess the relationship of patient-prescriber racial and ethnic concordance with receipt of an opioid prescription and patient-reported pain score at discharge.

**Results:**

Of 10,242 patients included in this analysis, 62.3% identified as NHW, 19.1% Hispanic, 9.7% NHB, and 8.9% Asian. About half (52.8%) of patients were discharged by a racially and ethnically concordant prescriber. Patient-prescriber racial and ethnic concordance was not associated with receipt of an opioid prescription (adjusted odds ratio [aOR] 0.82, confidence interval [95% CI] 0.67–1.00) or reporting a pain score ≥5 (aOR 0.90, 95% CI 0.69–1.16). However, NHB and Hispanic patients were less likely to receive an opioid prescription (aOR 0.73, 95% CI 0.56–0.95; aOR 0.73, 95% CI 0.57–0.92, respectively) and significantly more likely to report a pain score ≥5 (aOR 2.13, 95% CI 1.51–3.00; aOR 1.48 95% CI 1.08–2.01, respectively) than NHW patients, even when accounting for concordance.

**Conclusion:**

Disparities in postpartum opioid prescribing and pain perception are not ameliorated by patient-prescriber racial and ethnic concordance.

## Introduction

Data have consistently shown that patients of racial and ethnic minority groups receive less opioid pain treatment compared to non-Hispanic white (NHW) patients.^1–5^ Notably, recent data have shown that racial and ethnic disparities in postpartum opioid prescribing exist.^5,6^ Despite postpartum patients of Hispanic and non-Hispanic black, (NHB) racial and ethnic identity being more likely to report higher pain scores, they are significantly less likely to receive an opioid prescription on hospital discharge than their NHW counterparts.^5^ While these disparities are well documented, the underlying mechanisms are likely complex and multifactorial.^1,7^

Patient-provider racial and ethnic concordance, defined as the patient and provider sharing racial and ethnic identity, has been associated with improved health outcomes and utilization.^8–13^ For instance, patient-provider racial concordance has been associated with longer clinic interactions, increased patient satisfaction, increased adherence to antibiotic prescribing guidelines, and reduced newborn mortality.^9–14^ A systematic review found that racial concordance was associated with better outcomes in the communication domains of satisfaction, information giving, partnership building, participatory decision-making, visit length, supportiveness, and respect of conversations.^8^

Specifically in reference to pain, a recent study using simulated clinical interactions found that patient-provider racial and ethnic concordance resulted in significantly lower pain levels and pain-induced physiologic arousal among NHB participants.^12^ It remains unknown whether these results would translate to differences in real clinical settings.

Thus, the aim of this study was to evaluate whether patient-prescriber racial and ethnic concordance is associated with postpartum opioid prescribing and patient-reported pain scores.

## Materials and Methods

This is a retrospective cohort study of patients hospitalized for delivery at a high-volume, academic, tertiary care center from December 1, 2015 to November 30, 2016. This was a planned secondary analysis of an observational study, in which the primary aim was to describe opioid prescribing patterns among postpartum individuals.^15^

At this institution, there are over 11,500 deliveries annually. More than 200 private and university-employed attending physicians, nurse practitioners, midwives, and trainee physicians care for patients. Trainee physicians include obstetrics and gynecology residents and maternal-fetal medicine fellows. Advanced practitioners were defined as nurse practitioners and certified nurse–midwives. Practice groups include both private and university-employed providers and range in size from solo practitioners to 14-member groups. Groups include a mix of attending physicians and advanced practitioners.

The obstetrical team typically manages postpartum pain. In some groups, individual providers will continue to see patients whom they have delivered; in other groups, that responsibility may be shared among providers. Trainee physicians are primarily involved in the postpartum care of patients who received antenatal care in the hospital-based clinics at this institution. While trainee physicians are supervised by university-employed attending physicians, they independently write discharge prescriptions.

Trainee physicians are not universally involved in all deliveries, nor are they universally involved in the postpartum care of all patients. Although trainee physicians sometimes do assist in the postpartum care of patients not meeting the conditions described previously, each private health care provider group is responsible for discharge and prescribing of their group's patients. In addition, no hospital guidelines about postpartum narcotic prescriptions for either inpatient or outpatient use existed during the study period.

Postpartum patients were included in this analysis if they were 18 years of age or older, did not have a documented allergy to nonsteroidal anti-inflammatory drugs or morphine, self-identified as NHW, NHB, Hispanic, or Asian race and ethnicity, and were discharged by a prescriber who self-identified as NHW, NHB, Hispanic, or Asian race and ethnicity. Due to the inability to assess racial and ethnic concordance, patients and prescribers of “other” or “unreported” racial or ethnic groups were excluded. Patients with a diagnosed opioid use disorder, a prescription for buprenorphine, methadone, or fentanyl within 1 year of hospitalization, and those with explicit evidence of recent opioid use, defined as having received three or more prescriptions for an opioid in the year before delivery, were excluded.

Prior opioid prescriptions were identified through electronic medical records (EMR). Finally, we excluded patients who received general anesthesia, were admitted to the intensive care unit, had a hysterectomy, or had an inpatient postpartum hospitalization exceeding 10 days, because these rare events may alter analgesic requirements.^16^

EMRs were queried for demographic, clinical, and pharmacy data. Demographic data included maternal age, racial and ethnic identity, marital status, and insurance status. Patients have the option to select their racial and ethnic identity via the patient portal of the EMR or, if no answer is populated at the time of admission, are queried on admission. Patient clinical data obtained included history of substance use disorder, psychiatric conditions, body mass index (BMI) at delivery, mode of delivery, and patient-reported pain score before hospital discharge.

In addition, the presence of obstetrical complications was abstracted, including infectious complications (e.g., chorioamnionitis, endometritis, and wound and perineal infection), postpartum hemorrhage (estimated blood loss of ≥1000 mL), and major vaginal laceration (third or fourth degree vaginal laceration). Nurses assess pain scores at every vital sign evaluation; patients are asked to report their pain on a scale of 0 to 10, with 0 equating no pain and 10 equating the worst pain imaginable. EMRs were also used to determine the discharge prescriber and whether an opioid prescription was provided at discharge.

Employee administrative databases were queried for prescriber data, including prescriber training level, self-reported gender, and racial and ethnic identity. Prescriber training was categorized as attending physician, advanced practitioner, or trainee physician. Gender was classified as male or female. Patient-prescriber pairs were categorized as racially and ethnically concordant if they shared the same racial and ethnic identity and will hereafter be referred to as “concordant” for simplicity. The discharge prescriber was selected as the provider of interest because this individual made the decisions regarding prescribing and, based on common practice at this institution, was commonly the primary postpartum provider.

The primary outcomes were (1) the receipt of an opioid prescription at postpartum hospital discharge and (2) patient-reported pain score at the assessment most proximal to discharge, which we dichotomized as scores <5 and scores greater ≥5. Pain scores were dichotomized at this point because a score of 5 or greater generally represents pain requiring analgesia for treatment.^17^ The pain score immediately before discharge was chosen, as it is the one which should have the greatest influence on discharge prescribing, if prescribing is individualized to need.

We performed bivariable comparisons to identify differences in patient and prescriber demographic characteristics and clinical factors associated with patient-prescriber racial and ethnic concordance. Multivariable logistic regression models with a random effect term to account for clustering by provider were used to estimate the association of concordance with receipt of an opioid prescription and patient-reported pain score greater ≥5.

To evaluate whether any observed association with opioid prescribing or pain score differed by route of delivery, interaction terms between concordance and vaginal delivery were entered into the multivariable regression, and retained if they were significant, with the plan to perform additional stratified analyses by route of delivery for significant outcomes. Covariates for which *p*<0.05 on bivariable analysis and had been hypothesized before analyses to be potential confounders were evaluated in the multivariable models.

A *p*-value of <0.05 was used to define statistical significance, and all tests were two-tailed. Statistical analyses were performed with Stata v.16 (StataCorp, College Station, TX). This study was approved by the Institutional Review Board at Northwestern University. Strengthening the Reporting of Observational Studies in Epidemiology guidelines for observational research were followed.^18^

## Results

**FIG. 1.**
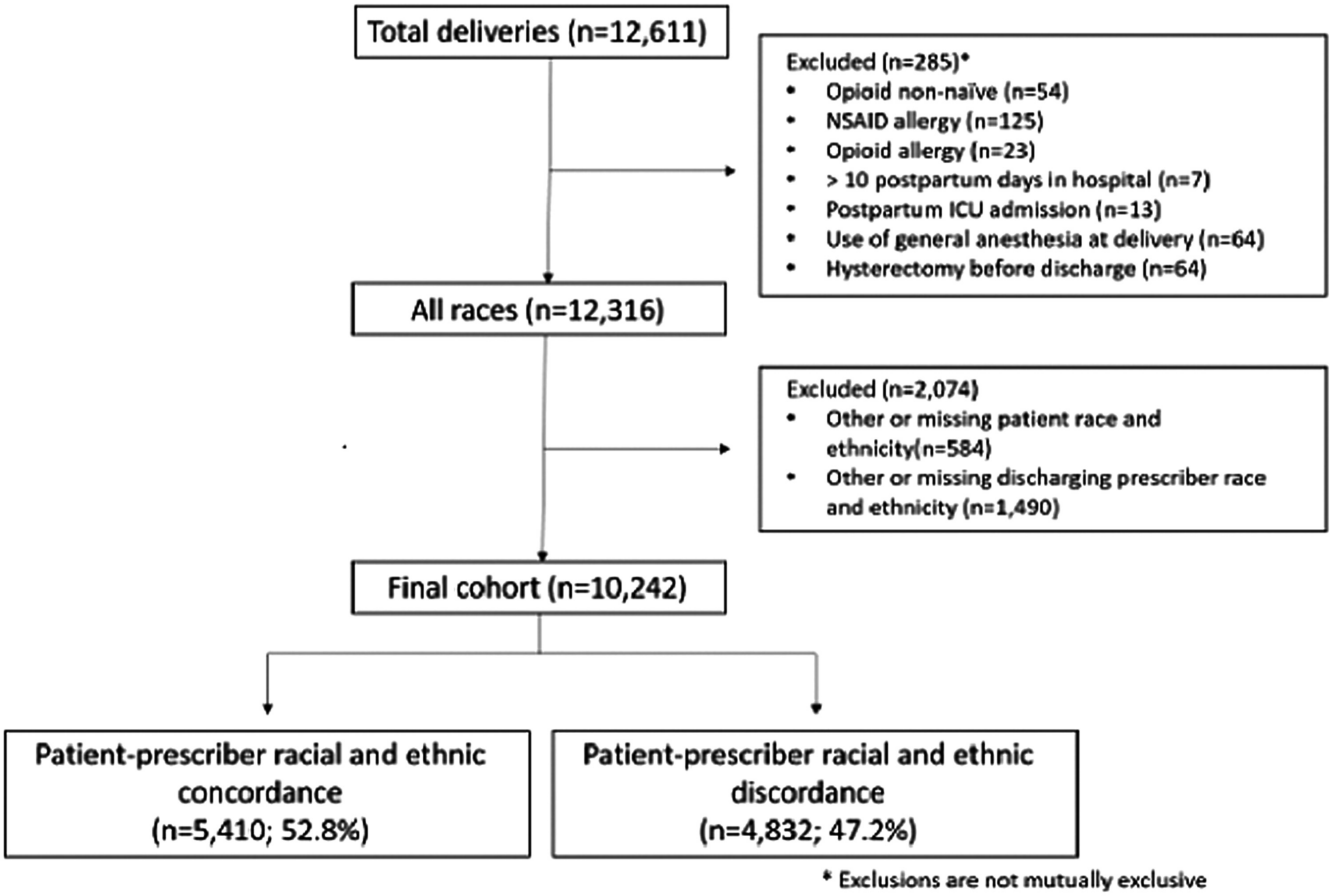
Cohort flowchart.

Of 12,611 individuals who gave birth in the study period, 10,242 were eligible for inclusion (Fig. 1). More than half (*n*=5410; 52.8%) of patients were cared for by a concordant prescriber. The majority (62.3%) of patients self-identified as NHW, while 9.7% self-identified as NHB, 19.1% as Hispanic, and 8.9% as Asian.

Patients cared for by a concordant prescriber were significantly older (33.2±4.1 vs. 31.4±5.4; *p*<0.001), more likely to be NHW (92.7% vs. 28.1%; *p*<0.001), and married (86.0% vs. 66.6%; *p*<0.001) than patients cared for by a discordant provider. They were less likely to have public insurance (40.5% vs. 56.5%; *p*<0.001) and to experience an infectious complication (6.8% vs. 7.9%; *p*=0.036). In addition, patients cared for by concordant prescribers were more likely to have a history of tobacco use (10.7% vs. 9.1%; *p*=0.007), depression (13.4% vs. 10.2%; *p*<0.001), or anxiety (14.5% vs. 10.4%; *p*<0.001), and to have a lower BMI (29.6±5.1 kg/m^2^ vs. 31.1±6.0 kg/m^2^; *p*<0.001) than patients cared for by discordant prescribers.

Patients with concordant prescribers were more likely to be discharged by an attending physician (70.8% vs. 63.6%; *p*<0.001) and less likely to be discharged by a female prescriber (84.9% vs. 89.7%; *p*<0.001) than patients with discordant prescribers (Table 1).

**Table 1.** Patient Characteristics by Concordant Versus Discordant Status with Provider

	Patient-prescriber racial and ethnic concordance	Patient-prescriber racial and ethnic discordance	*p*
	*N* =5410	*N* =4832	
Patient characteristics			
Age, years	33.2±4.1	31.4±5.4	<0.001
Race and ethnicity			
NHW	5017 (92.7)	1358 (28.1)	<0.001
NHB	102 (1.9)	903 (18.7)	
Hispanic	175 (3.2)	1775 (36.7)	
Asian	116 (2.1)	796 (16.5)	
Body mass index, kg/m^2^	29.6±5.1	31.1±6.0	<0.001
Nulliparous	2792 (51.6)	2271 (47.0)	<0.001
Married^a^	4579 (86.0)	3180 (66.6)	<0.001
Public insurance^b^	2160 (40.5)	2688 (56.5)	<0.001
History of tobacco use^c^	568 (10.7)	429 (9.1)	0.007
History of substance abuse	33 (0.61)	29 (0.60)	0.949
History of depression	726 (13.4)	495 (10.2)	<0.001
History of anxiety	784 (14.5)	505 (10.4)	<0.001
Vaginal delivery	4007 (74.1)	3521 (72.9)	0.170
Major vaginal laceration^d,e^	125 (3.1)	93 (2.6)	0.215
Postpartum hemorrhage^f^	173 (3.2)	168 (3.5)	0.432
Infectious complications^g^	367 (6.8)	380 (7.9)	0.036
Prescriber characteristics			
Female discharge prescriber	4594 (84.9)	4333 (89.7)	<0.001
Training of discharge prescriber			
Trainee physician	128 (2.4)	651 (13.5)	<0.001
Advanced practioner	1452 (26.8)	1107 (22.9)	
Attending physician	3830 (70.8)	3074 (63.6)	

Data presented as *N* (%) or mean±standard deviation.

^a^ Total *N*=10,097.

^b^ Total *N*=10,092.

^c^ Total *N*=10,074.

^d^ Total *N*=7531.

^e^ Major vaginal laceration is defined as third or fourth degree lacerations.

^f^ Postpartum hemorrhage was defined as an estimated blood loss of ≥1000 mL.

^g^ Infectious complications include chorioamnionitis, endometritis, wound, and perineal infection.

NHB, non-Hispanic black; NHW, non-Hispanic white.

During the study period, there were 216 prescribers (Table 2). The majority of prescribers self-identified as NHW (*N*=131, 60.6%), female (*N*=183, 84.7%), and were attending physicians (*N*=126, 58.3%) (Table 2).

**Table 2.** Prescriber Characteristics

	*N*=216
Prescriber characteristics	*N* (%)
Race and ethnicity	
NHW	131 (60.6)
NHB	14 (6.5)
Hispanic	41 (19.0)
Asian	30 (13.9)
Gender	
Female	183 (84.7)
Prescriber training	
Attending physician	126 (58.3)
Advanced practitioner	40 (18.5)
Trainee	50 (23.1)

Almost half of patients (44.9%, *N*=4,557) received an opioid prescription at discharge. In bivariable analyses, there was no significant difference in whether an opioid prescription was received by patient-prescriber concordance (52.2% vs. 47.8%; *p*=0.28) (Table 3). In multivariable analysis, there was no significant difference in the odds of receiving an opioid prescription at discharge by patient-prescriber concordance (adjusted odds ratio [aOR] 0.82, confidence interval [95% CI] 0.67–1.00).

**Table 3.** Odds of Receiving an Opioid Prescription, by Patient-Prescriber Racial and Ethnic Concordance^a^ and Patient Race and Ethnicity

	Opioid prescription (*N*=4557)	No opioid prescription (*N*=5685)	*p*	OR (95% CI)	aOR (95% CI)^b^
Race and ethnic concordance					
Yes	2380 (52.2)	3030 (53.3)	0.281	0.96 (0.86–1.07)	0.82 (0.67–1.00)
No	2177 (47.8)	2655 (46.7)		1.00 (Ref.)	1.00 (Ref.)
Patient race and ethnicity					
NHW	2943 (64.6)	3432 (60.4)	<0.001	1.00 (Ref.)	1.00 (Ref.)
NHB	453 (9.9)	552 (9.7)		1.02 (0.86–1.20)	0.73 (0.56–0.95)
Hispanic	750 (16.5)	1200 (21.1)		0.92 (0.80–1.06)	0.73 (0.57–0.92)
Asian	411 (9.0)	501 (8.8)		0.92 (0.79–1.08)	0.79 (0.62–1.00)

^a^ Racial and ethnic concordance: defined as a patient and prescriber pair that share the same racial and ethnic identity.

^b^ Multivariable logistic regression accounting for racial and ethnic concordance, patient race and ethnicity, marital status, nulliparity, insurance, body mass index, vaginal delivery, infectious complications, history of tobacco use, depression, or anxiety, prescriber training, and clustering by prescriber.

aOR, adjusted odds ratio; CI, confidence interval.

However, even after accounting for concordance, patient NHB and Hispanic racial and ethnic identity remained significantly associated with receiving an opioid prescription at discharge. Specifically, compared to NHW patients, NHB and Hispanic patients were significantly less likely to receive an opioid prescription at discharge (aOR 0.73, 95% CI 0.56–0.95 and aOR 0.73, 95% CI 0.57–0.92, respectively).

A small proportion, or 5.6% (*N*=570), of patients reported a pain score ≥5 at discharge. On bivariable analyses, patients with a concordant prescriber were significantly less likely to report a pain score ≥5 than patients with a discordant prescriber (39.3% vs. 60.7%; *p*<0.001; Table 4). However, in the multivariable analysis, patient-prescriber concordance did not remain significantly associated with patient-reported pain score ≥5 (aOR 0.90, 95% CI 0.69–1.16), but NHB and Hispanic patients were significantly more likely to report a pain score ≥5 at discharge (aOR 2.13, 95% CI 1.51–3.00 and aOR 1.48, 95% CI 1.08–2.01, respectively) compared to NHW patients.

**Table 4.** Patient-Reported Pain Score, by Patient-Prescriber Racial and Ethnic Concordance^a^ and Patient Race and Ethnicity

	Pain score ≥5 (*N*=570)	Pain score <5 (*N*=9,672)	*p*	OR (95% CI)	aOR (95% CI)^b^
Racial and ethnic concordance					
Yes	224 (39.3)	5186 (53.6)	<0.001	0.58 (0.48–0.70)	0.90 (0.69–1.16)
No	346 (60.7)	4486 (46.4)		1.00 (Ref.)	1.00 (Ref.)
Patient race and ethnicity					
NHW	255 (44.7)	6120 (63.3)	<0.001	1.00 (Ref.)	1.00 (Ref.)
NHB	118 (20.7)	887 (9.2)		3.03 (2.38–3.86)	2.13 (1.51–3.00)
Hispanic	143 (25.1)	1807 (18.7)		1.82 (1.45–2.28)	1.48 (1.08–2.01)
Asian	54 (9.5)	858 (8.9)		1.50 (1.10–2.03)	1.43 (1.00–2.05)

^a^ Race and ethnic concordance: defined as a patient and provider pair that share the same racial and ethnic identity.

^b^ Multivariable logistic regression accounting for racial and ethnic concordance, patient race and ethnicity, marital status, nulliparity, insurance, body mass index, vaginal delivery, infectious complications, history of tobacco use, depression or anxiety, prescriber training, and clustering by prescriber.

aOR, adjusted odds ratio; CI, confidence interval.

Interaction terms between patient-prescriber racial and ethnic concordance and route of delivery with postpartum opioid prescription or patient-reported pain score ≥5 (respective aOR 1.13, 95% CI 0.88–1.46; aOR 1.12, 95% CI 0.81–1.56) were not significant.

## Discussion

In this retrospective cohort study, patient-prescriber racial and ethnic concordance was not associated with receipt of a postpartum opioid prescription or a patient-reported pain score of ≥5 at discharge. Compared to NHW patients, NHB and Hispanic patients had 27% decreased odds of receiving an opioid prescription at postpartum discharge, even after accounting for patient-prescriber racial and ethnic concordance. Compared to NHW patients, NHB and Hispanic patients had 113% and 48% increased odds, respectively, of reporting a pain score ≥5 at discharge, even after accounting for patient-prescriber racial and ethnic concordance. Findings did not differ by route of delivery.

Literature suggests that racial and ethnic minority patients experience poorer health outcomes and communication with their provider, measured by metrics such as satisfaction, length of visit, and participatory decision-making, than their NHW counterparts.^8^ Racial and ethnic disparities also manifest among obstetric outcomes.^5,19^ Prior literature has shown that patients who identify as NHB or Hispanic receive fewer morphine milligram equivalents during their postpartum hospitalization and are less likely to receive an opioid prescription at discharge, when compared to NHW patients.^5,6^

Although some have theorized that this disparity may be diminished in the setting of care by providers who themselves identify as minorities, our findings indicate that this effect remains present in postpartum opioid prescribing and reported pain, even when accounting for patient-prescriber concordance.

It is worth noting that previous studies have demonstrated that patient-provider concordance may mitigate some disparities in health outcomes.^9–14^ A systematic review identified improvement in aspects of communication, such as satisfaction, partnership building, and participatory decision-making, when patients and physicians shared the same racial identity.^8^ In addition, studies of concordance of patients and providers with regard to other personal attributes have suggested potential benefits; for example, patient-provider gender concordance has been associated with improved health outcomes, such as cancer screening and patient satisfaction.^20,21^ Patients who share characteristics with their providers may feel more comfortable and have improved communication with their provider, potentially leading to improved health outcomes.

Similarly, providers who share characteristics with their patients may have a greater understanding of patients' sociocultural contexts, which may result in improved health outcomes. Thus, it is possible that patient-provider concordance may alleviate the impact of racial or ethnic disparities in some settings. Nevertheless, we did not demonstrate an effect in our analysis; in this specific obstetric context, concordance was not associated with postpartum opioid prescribing or reported pain.

Racial and ethnic disparities in health are thought to occur at three levels: patient, provider, and system.^22^ Our data contribute to our understanding of racial and ethnic disparities in maternal health by examining the level at which patients and providers interface. The presence of inequity in prescribing despite patient-prescriber concordance highlights the complexity and multifactorial nature of racial and ethnic disparities. Our findings indicate the role of a more global effect, such as that of structural or systemic differential treatments by race and ethnicity, rather than solely at the level of the individual provider, which warrant further investigation.

Both implicit and explicit racial and ethnic biases have potential impacts on patients' health; in fact, studies have found that patient experiences of racism are associated with delaying seeking care and being less adherent to treatment regiments.^23^ Future research may explore provider bias in postpartum opioid prescribing and subsequently test effective methods to increase provider awareness of potential biases.

One mechanism of minimizing the clinical impact of differences in postpartum pain management is increasing standardization and consistency of pain management and opioid prescribing protocols. By reducing individual prescriber variability, standard protocols may decrease racial and ethnic disparities. While interventions using tiered order sets and shared decision-making have been successful at reducing postpartum opioid use and prescribing,^7,24^ further work is necessary to investigate if the effect of such interventions resulted in improvement in racial and ethnic disparities in opioid pain management.

Moreover, it is important to acknowledge that the goal should not be universal decreases in opioid prescribing, but rather increases in patient satisfaction and adequacy of pain control; optimization of the postpartum experience may actually warrant increased opioid prescribing for some patients whose pain has not been adequately controlled.

There is notable homogeneity in prescriber race and ethnicity in our study population, as the majority identified as NHW; this circumstance is reflective of the health care workforce across the country. According to a study on the obstetrician and gynecologist workforce from the American College of Obstetricians and Gynecologists, NHB and Hispanic providers are underrepresented, only accounting for 11.1% and 6.7%, respectively, of all OB/GYN providers in the United States.^25^

The American Medical Association and the Association of American Medical Colleges have expressed the need to prioritize greater diversity in the physician workspace as a means to combat structural and systemic racial and ethnic disparities.^26,27^ For example, data from the National Bureau of Economic Research showed that increasing the overall number of NHB cardiovascular doctors can reduce the NHW-NHB gap in cardiovascular mortality by 19%.^28^

The relationship between increasing proportions of underrepresented minority individuals in health care and improvements in health outcomes has been demonstrated across other fields.^29,30^ Although our data suggest no effect of concordance, this analysis warrants replication in the future, when greater diversity of prescribers may exist.

Our study has limitations. First, we only included deliveries at an urban, medical center in the Midwest, which may not be generalizable to other areas of the country. The retrospective nature of this study makes it susceptible to incomplete data and misclassification. In addition, while over half of the included patients had a racially and ethnically concordant prescriber, this was largely driven by NHW patient-prescriber dyads. The majority of NHB, Hispanic, and Asian patients, therefore, did not have a racially and ethnically concordant prescriber. This may limit our ability to identify differences among minority race and ethnicities and to assess how greater prescriber diversity may alter associations.

Furthermore, we were unable to analyze patients who identify as mixed racial and ethnic identity or those self-identifying outside of the analyzed groups. In addition, we are unable to evaluate patient preferences, which may be driven by cultural preferences or other unmeasurable factors, and could further contextualize the results.

Cultural and community differences regarding expectations of pain and pain management are timely and important questions for future research. This study was conducted in from December 1, 2015 to November 30, 2016 and the impact of temporal changes in opioid prescribing is not known. Finally, our study defined prescriber as the discharging prescriber, which may not be the individual with whom the patient interacted the most during their care. This is a potential source of unobserved confounding since some patients may have had a predetermined relationship with their discharging prescriber, while others may have only interacted with this prescriber inpatient.

However, if postpartum discharge prescriptions were truly individualized, the discharge prescriber would have the greatest influence on the receipt of an opioid prescription and patient-reported pain score closest to discharge.

A major strength of this study is the novel examination of patient-prescriber racial and ethnic concordance as a potential mechanism influencing racial and ethnic differences in postpartum opioid prescriptions. Previous research has examined mechanisms contributing to racial and ethnic disparities in opioid use and prescription,^2,4,31^ but minimal attention has been applied to postpartum opioid prescribing in the postpartum population.

Further, most current literature examining racial and ethnic concordance exclusively included attending physicians in their analysis,^8–14^ but our analysis also includes trainee physicians and advanced practitioners, who also play a major role in patient care. Given that prescriber training level may be associated with administration of opioid prescriptions at postpartum discharge, these types of providers are important to include in disparity analyses.^32^ Finally, this study used a large dataset with high-quality clinical and administrative data.

In summary, we evaluated the association of patient-prescriber racial and ethnic concordance with the receipt a postpartum opioid prescription and patient-reported pain score at discharge. No significant association was found by patient-prescriber concordance, yet, racial and ethnic inequities in these outcomes persisted. Further work examining mechanisms underlying these disparities in postpartum pain management is warranted.

## Abbreviations Used

aOR: adjusted odds ratio
BMI: body mass index
CI: confidence interval
EMR: electronic medical records
NHB: non-Hispanic black
NHW: non-Hispanic white

## References

[1] Mossey JM. Defining racial and ethnic disparities in pain management. Clin Orthop Relat Res. 2011;469:1859–1870. 21249483 10.1007/s11999-011-1770-9 PMC3111792

[2] Pletcher MJ, Kertesz SG, Kohn MA, et al. Trends in opioid prescribing by race/ethnicity for patients seeking care in US emergency departments. JAMA. 2008;299:70–78. 18167408 10.1001/jama.2007.64

[3] Rust G, Nembhard WN, Nichols M, et al. Racial and ethnic disparities in the provision of epidural analgesia to Georgia Medicaid beneficiaries during labor and delivery. Am J Obstet Gynecol. 2004;191:456–462. 15343221 10.1016/j.ajog.2004.03.005

[4] Friedman J, Kim D, Schneberk T, et al. Assessment of racial/ethnic and income disparities in the prescription of opioids and other controlled medications in California. JAMA Intern Med. 2019;179:469–476. 30742196 10.1001/jamainternmed.2018.6721 PMC6450285

[5] Badreldin N, Grobman WA, Yee LM. Racial disparities in postpartum pain management. Obstet Gynecol. 2019;134:1147–1153. 31764723 10.1097/AOG.0000000000003561 PMC6905121

[6] Johnson JD, Asiodu IV, McKenzie CP, et al. Racial and ethnic inequities in postpartum pain evaluation and management. Obstet Gynecol. 2019;134:1155–1162. 31764724 10.1097/AOG.0000000000003505

[7] Holland E, Bateman BT, Cole N, et al. Evaluation of a quality improvement intervention that eliminated routine use of opioids after cesarean delivery. Obstet Gynecol. 2019;133:91–97. 30531571 10.1097/AOG.0000000000003010

[8] Shen MJ, Peterson EB, Costas-Muñiz R, et al. The effects of race and racial concordance on patient-physician communication: a systematic review of the literature. J Racial Ethn Health Disparities. 2018;5:117–140. 28275996 10.1007/s40615-017-0350-4 PMC5591056

[9] Greenwood BN, Hardeman RR, Huang L, Sojourner A. Physician-patient racial concordance and disparities in birthing mortality for newborns. Proc Natl Acad Sci U S A. 2020;117:21194–21200. 32817561 10.1073/pnas.1913405117 PMC7474610

[10] Morgan JR, Mari-Lynn D, Christiansen C, et al. Patient-provider race and sex concordance: new insights into antibiotic prescribing for acute bronchitis. J Health Dispar Res Pract. 2017;10:6.

[11] Street RL Jr., O'Malley KJ, Cooper LA, et al. Understanding concordance in patient-physician relationships: personal and ethnic dimensions of shared identity. Ann Fam Med. 2008;6:198–205. 18474881 10.1370/afm.821 PMC2384992

[12] Anderson SR, Gianola M, Perry JM, et al. Clinician-patient racial/ethnic concordance influences racial/ethnic minority pain: evidence from simulated clinical interactions. Pain Med. 2020. [Epub ahead of print]; DOI: 10.1093/pm/pnaa258. PMC8453614 32830855

[13] King WD, Wong MD, Shapiro MF, et al. Does racial concordance between HIV-positive patients and their physicians affect the time to receipt of protease inhibitors? J Gen Intern Med. 2004;19:1146–1153. 15566445 10.1111/j.1525-1497.2004.30443.x PMC1494794

[14] Frierson GM, Pinto BM, Denman DC, et al. Bridging the Gap: racial concordance as a strategy to increase African American participation in breast cancer research. J Health Psychol. 2019;24:1548–1561. 29172809 10.1177/1359105317740736

[15] Badreldin N, Grobman W, Chang K, et al. Opioid prescribing patterns among postpartum women. Am J Obstet Gynecol. 2018;219:103.e1–e8. 29630887 10.1016/j.ajog.2018.04.003 PMC6019620

[16] Grobman WA, Bailit JL, Rice MM, et al. Frequency of and factors associated with severe maternal morbidity. Obstet Gynecol. 2014;123:804–810. 24785608 10.1097/AOG.0000000000000173 PMC4116103

[17] Boonstra AM, Stewart RE, Köke AJ, et al. Cut-off points for mild, moderate, and severe pain on the numeric rating scale for pain in patients with chronic musculoskeletal pain: variability and influence of sex and catastrophizing. Front Psychol. 2016;7:1466. 27746750 10.3389/fpsyg.2016.01466 PMC5043012

[18] von Elm E, Altman DG, Egger M, et al. The Strengthening the Reporting of Observational Studies in Epidemiology (STROBE) Statement: guidelines for reporting observational studies. Int J Surg. 2014;12:1495–1499. 25046131 10.1016/j.ijsu.2014.07.013

[19] American College of Obstetricans and Gynecologists Committee on Healthcare for Underserved Women. Racial and ethnic disparities in obstetrics and gynecology. Obstet Gynecol. 2015;126:e130–e134. 26595584 10.1097/AOG.0000000000001213

[20] Malhotra J, Rotter D, Tsui J, et al. Impact of patient-provider race, ethnicity, and gender concordance on cancer screening: findings from Medical Expenditure Panel Survey. Cancer Epidemiol Biomarkers Prev. 2017;26:1804–1811. 29021217 10.1158/1055-9965.EPI-17-0660

[21] Rogo-Gupta LJ, Haunschild C, Altamirano J, et al. Physician gender is associated with Press Ganey patient satisfaction scores in outpatient gynecology. Womens Health Issues. 2018;28:281–285. 29429946 10.1016/j.whi.2018.01.001

[22] Jain JA, Temming LA, D'Alton ME, et al. SMFM Special Report: putting the “M” back in MFM: reducing racial and ethnic disparities in maternal morbidity and mortality: a call to action. Am J Obstet Gynecol. 2018;218:B9–b17. 10.1016/j.ajog.2017.11.591 29183819

[23] Ben J, Cormack D, Harris R, et al. Racism and health service utilisation: a systematic review and meta-analysis. PLoS One. 2017;12:e0189900. 29253855 10.1371/journal.pone.0189900 PMC5734775

[24] Rogers RG, Nix M, Chipman Z, et al. Decreasing opioid use postpartum: a quality improvement initiative. Obstet Gynecol. 2019;134:932–940. 31599842 10.1097/AOG.0000000000003512

[25] Orvos JM. ACOG releases new study on ob/gyn workforce. Contemporary OBGYN. 2017;July 2017 (Special Report).

[26] Jakubek K. New Policy Aimed at Increasing Diversity in Physician Workforce. Chicago, IL: American Medical Association: AMA, 2019.

[27] Darrell G. Kirch M. A Leadership Forum on Diversity and Inclusion. AAMC. Available at https://www.aamc.org/news-insights/insights/leadership-forum-diversity-and-inclusion Accessed June 25, 2021.

[28] Marcella Alsan OG, Grant Graziani. Does Diversity Matter for Health? Experimental Evidence from Oakland. National Bureau of Economic Research 2018 (NBER Working Paper Series). [Epub ahead of print]; DOI: 10.3386/w24787.

[29] Gomez LE, Bernet P. Diversity improves performance and outcomes. J Natl Med Assoc. 2019;111:383–392. 30765101 10.1016/j.jnma.2019.01.006

[30] Cohen JJ, Gabriel BA, Terrel C. The case for diversity in the health care workforce. Health Affairs. 2002;21:90–102. 12224912 10.1377/hlthaff.21.5.90

[31] Santoro TN, Santoro JD. Racial bias in the US opioid epidemic: a review of the history of systemic bias and implications for care. Cureus. 2018;10:e3733. 30800543 10.7759/cureus.3733 PMC6384031

[32] Badreldin N, Grobman WA, Chang KT, et al. Patient and health care provider factors associated with prescription of opioids after delivery. Obstet Gynecol. 2018;132:929–936. 30204691 10.1097/AOG.0000000000002862 PMC6153070

